# Optimising Concrete Crack Detection: A Study of Transfer Learning with Application on Nvidia Jetson Nano

**DOI:** 10.3390/s24237818

**Published:** 2024-12-06

**Authors:** C. Long Nguyen, Andy Nguyen, Jason Brown, Terry Byrne, Binh Thanh Ngo, Chieu Xuan Luong

**Affiliations:** 1 School of Engineering, University of Southern Queensland, Springfield, QLD 4300, Australia; long.nguyen@unisq.edu.au (C.L.N.); jason.brown2@unisq.edu.au (J.B.); 2 Academic Affairs Administration, University of Southern Queensland, Toowoomba, QLD 4350, Australia; terry.byrne@unisq.edu.au; 3 Faculty of Electrical and Electronic Engineering, University of Transport and Communications, Hanoi 100000, Vietnam; ngobinh74@utc.edu.vn (B.T.N.); chieu1256@utc.edu.vn (C.X.L.)

**Keywords:** Structural Health Monitoring, Artificial Intelligence, crack detection, concrete structures, transfer learning, Jetson Nano, digital image

## Abstract

The use of Artificial Intelligence (AI) to detect defects such as concrete cracks in civil and transport infrastructure has the potential to make inspections less expensive, quicker, safer and more objective by reducing the need for on-site human labour. One deployment scenario involves using a drone to carry an embedded device and camera, with the device making localised predictions at the edge about the existence of defects using a trained convolutional neural network (CNN) for image classification. In this paper, we trained six CNNs, namely Resnet18, Resnet50, GoogLeNet, MobileNetV2, MobileNetV3-Small and MobileNetV3-Large, using transfer learning technology to classify images of concrete structures as containing a crack or not. To enhance the model’s robustness, the original dataset, comprising 3000 images of concrete structures, was augmented using salt and pepper noise, as well as motion blur, separately. The results show that Resnet50 generally provides the highest validation accuracy (96% with the original dataset and a batch size of 16) and the highest validation F1-score (95% with the original dataset and a batch size of 16). The trained model was then deployed on an Nvidia Jetson Nano device for real-time inference, demonstrating its capability to accurately detect cracks in both laboratory and field settings. This study highlights the potential of using transfer learning on Edge AI devices for Structural Health Monitoring, providing a cost-effective and efficient solution for automated crack detection in concrete structures.

## 1. Introduction

Since the last century, many sophisticated civil and transport structures have been built due to the rapid development of efficient design and construction methodologies. Examples include high-rise buildings, dams, bridges, tunnels and public/utility infrastructure. However, the performance and functionality of these important structures can be weakened because of subsidence, improper usage, ageing components and materials. As a result, servicing and maintaining the function and integrity of these structures is important, which has led to the creation of a new consideration in the civil and construction field called Structural Health Monitoring (SHM). Inspection and monitoring can help owners and engineers classify the abnormal behaviour of structures under specified conditions based on several assessments and standards.

Infrastructure inspection is usually performed by certified specialists using equipment and best practise assessment techniques. In the early 1980s, building condition assessments were conducted just by visual inspection and simple tools such as hammers and hearing equipment. Over time, this task has been improved with the support of not only various electronic devices but also assessment standards and guidelines. A simple example of the latter is the Australian standard for the inspection of buildings, AS 4349.1-1995, which focuses on residential buildings [1]. Alternatively, the inspection is commonly performed using Non-Destructive Testing (NDT) techniques, including ground-penetrating radar, laser scanning, thermography, ultrasound and close-range photogrammetry [2]. Once the field tests are completed, the test data are reviewed and combined with the inspector’s opinion to make maintenance decisions.

Despite the popularity of human-based inspection, the application of this approach has several significant limitations when applied to large civil structures, such as bridges, high-rise buildings or dams. The first limitation is the labour cost and time this approach requires qualified inspectors to spend in order to inspect large structures [3]. In recent years, and certainly during the COVID-19 pandemic, the skilled labour shortage has been one of the most problematic issues for most industries, and their heavy reliance on human specialists can result in overdue infrastructural inspections and the costly maintenance that follows them. Furthermore, for many large-scale structures, the inspectors are often left to deal with difficult locations requiring expensive scaffoldings or special equipment like scissor lifts to gain access [4]. Finally, one of the most important limitations of human inspection is making incorrect decisions depending upon the opinion of a human assessor, who may make errors or be subjected to peer pressure. As a result, human-based inspection can result in elevated risk and inefficiency.

Since the late 20th century, applications of modern SHM techniques have become popular in civil engineering and construction. A modern SHM system provides efficient methods to evaluate and monitor the health and performance of the structures without the need for human intervention during the monitoring or inspection process. This kind of system can help engineers automate the process of damage identification, including diagnosis and prognosis of the changes in a structure under specified conditions. Among various modern SHM techniques, the most popular and effective ones are response-based and computer vision-based [5]. The response-based method monitors the behaviours of structures based on their dynamic properties and physical characteristics, which are measured by temporary or permanent contact sensors in real time [6]. To monitor large structures at a sufficient resolution, the response-based method might require a considerable number of contact sensors embedded throughout the structure. The main limitation of this approach is it often increases capital, installation and operating costs.

The computer vision-based method, on the other hand, utilises vision sensors to collect responses and other information about the structure in a remote manner, meaning they can be easily reused with minimum increases in costs. Vision sensors are inexpensive and available in many forms, such as digital cameras, laser scanners and optical lenses [5]. In this way, inspection data such as images or videos can be acquired from a distance, which greatly minimises the risks for human inspectors in dealing with difficult locations. In recent years, with the efforts from both academia and industry, unmanned aerial vehicles (UAVs or drones) have begun to be utilised widely for complicated assessment tasks such as bridge inspection. Whilst the initial purpose of this application was to ease the image data acquisition process, this vision-based technology offers real potential for the rapid monitoring and automated inspection of civil and transport infrastructure.

There are several studies and real-life projects that have been using the vision-based method to assess the structural conditions in dams [7], tunnels [8], highways [9], pavements [10], railways [11], concrete buildings [12], steel buildings [13], pipelines [14] and so forth. These projects have utilised vision sensors to capture digital image information and post-process them for condition assessment with the support of Artificial Intelligence (AI). A further review of these applications shows that convolutional neural networks (CNNs), a kind of modern AI algorithm for image classification, have been commonly used in the context of centralised or cloud-based systems where computations are executed in the direct support of a powerful graphical processing unit (GPU). However, there is no similar research or studies on the assessment performance of CNNs, which automatically identify structural defects when installed in Edge computing embedded devices. The research in this paper focuses on evaluating the performance of several common CNNs using transfer learning to find the best-performing network for integration with Edge AI devices, such as the NVIDIA Jetson Nano, for the autonomous detection of multiple cracks on concrete surfaces in real time. The term “Edge AI device” refers to a device that is both sourcing data (i.e., images in this case) and performing AI predictions to determine what is represented in the data without necessarily transferring the raw data to another location for analysis.

We discuss the Edge AI devices and CNN models in more detail in Section 2. However, at this point, it is worth noting that there are various computer vision techniques that can be employed depending on the objective, as illustrated in Table 1. Image classification is the most basic technique, in which the objective is to predict the class(es) or type(s) of object(s) in an image without understanding the exact location, size and shape of the object(s). For example, in the context of our research, we would use image classification to determine whether or not a crack exists in a concrete structure depicted in an image. Object detection and semantic segmentation can be viewed as more complex refinements of image classification, in which we are interested in predicting not just the class of an object in an image but also the location of the object in the image (object detection) and its pixel boundaries (semantic segmentation). For this initial investigation into employing computer vision techniques on Edge AI devices for crack detection, we therefore focus on image classification in the remainder of this paper.

To evaluate the deployment of transfer learning algorithms on Edge AI devices, this paper examines the performance of six CNNs for crack detection on concrete surfaces, namely Resnet18, Resnet50, GoogLeNet, MobileNetV2 and MobileNetV3 (small and large). To enhance the robustness of the training model, as well as to simulate the actual environmental conditions, three different noise patterns, salt and pepper, motion blur and a combination of salt and pepper and motion blur, are applied onto an original concrete dataset to generate three additional datasets for training purposes. Furthermore, the CNN model with the best classification performance is employed in the Jetson Nano for inference testing in the field.

## 2. Background and Related Work

### 2.1. Artificial Intelligence and Its Subfields

**Figure 1:**
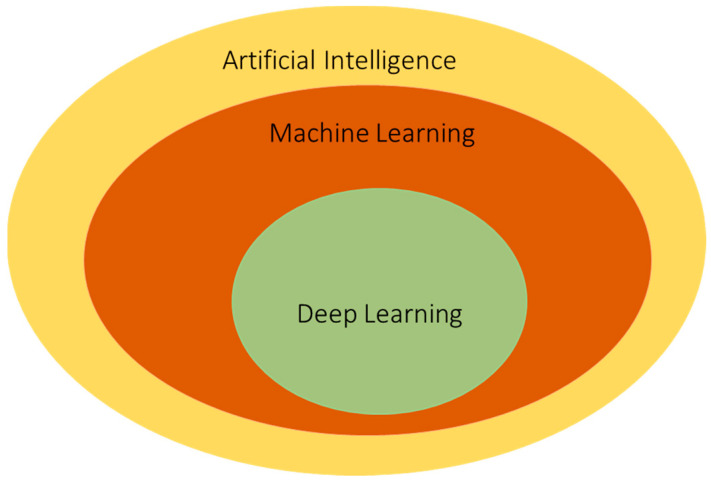
The subfields of Artificial Intelligence.

Artificial Intelligence (AI) first began in the 1950s for solving complex mathematical problems and developed significantly in the early 2000s. Machine Learning (ML), a subfield of AI, has played a vital role in the process of collecting and analysing a great quantity of data when engineers and researchers monitor structural health. ML extracts the features and patterns of the images or videos and then enters them into Artificial Neural Network (ANN) algorithms to classify these features. However, ML and ANN algorithms often require experts to monitor and extract the features manually, which can be a limitation for engineers when dealing with huge data loads and complicated features [15,16]. Thus, deep learning (DL) was developed to solve this problem. In contrast to basic ML techniques, DL has multiple hidden layers and can extract numerous complex patterns from data automatically. Accordingly, the accuracy of DL is always higher than ML when dealing with complex and substantial amounts of data [6,17]. Figure 1 shows the subfields of Artificial Intelligence.

The DL model would appear to be the most suitable solution for defect detection in the SHM system. However, training a DL network from scratch can take significant time, require a massive dataset of images and require a powerful computational device. As a result, transfer learning (TL) with convolutional neural networks (CNNs) has been deployed for training defect detection to reduce training time and enhance accuracy. The weights and layers from the pre-trained models are transferred to another untrained network with the new dataset and re-trained as a new model. The benefits of utilising TL include reducing the number of datasets, both labelled and unlabelled, and improving efficiency when training a new model. For instance, the TL technique has been utilised in various CNN models to classify and segment cracks on masonry surfaces [18]. As a result, the performance of crack inspection tasks is leveraged significantly on both patch and pixel levels. On the other hand, another study proposed Step Transfer Learning with an Extreme Learning Machine (STELM) for automated concrete crack detection [19]. This approach incorporates double-step transfer learning, where two separate Resnet models are re-trained and fine-tuned on different datasets. The features extracted from these models are concatenated and subsequently used to classify concrete cracks.

AI and its subfield models are often operated and trained on servers or workstation systems, which consist of multiple powerful computers, with connections via the Internet and cloud systems. This has brought many benefits in the processing and characterisation of large volumes of data. However, concerns about the latency and security of AI based on the cloud have been raised [20,21]. As a result, Edge computing has been introduced with a distributed computing paradigm, which can help the AI models to operate on a local device with a processor unit. The combination of AI and Edge computing has generated Edge AI, which can reallocate computations and processes on the cloud to the local hardware. Therefore, the amount of data transmission can be reduced as well as the data latency. Recently, with the ongoing development of computing hardware, embedded devices have been introduced with powerful functionality to support AI training and Edge computing.

### 2.2. Embedded Device

**Figure 2:**
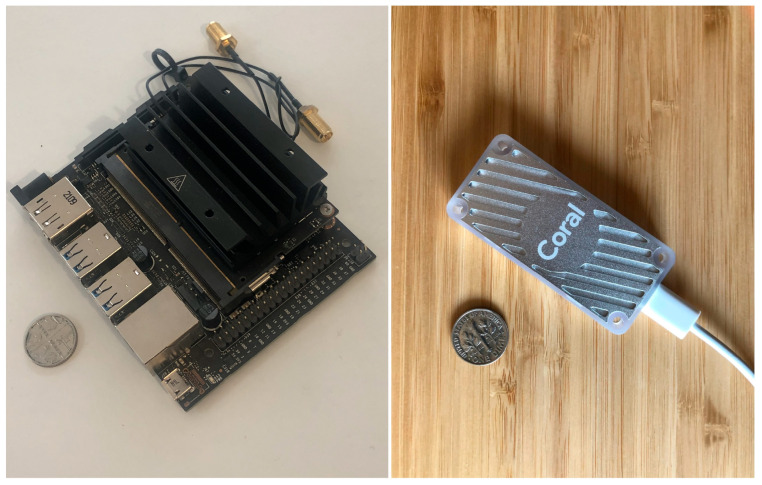
Two popular embedded devices, including Nvidia Jetson Nano (**left**) and Google Coral USB accelerator (**right**), with Edge AI capacity [23,24].

Embedded devices are compact computers with microprocessor-based hardware and possibly Edge AI hardware accelerators. Hardware acceleration boosts the speed and performance of deep learning tasks, offering greater scalability, reliability and security for the information. Major tech companies, such as Google and Nvidia, have brought to the market popular products such as Coral USB Accelerator and Jetson Nano (shown in Figure 2), which both have the same Edge AI focus. Google Coral is a USB stick with an Edge TPU, optimised for running models with ultra-low power consumption and high efficiency. However, the Coral USB Accelerator is just a USB booster and might require additional computers for training and testing. On the other hand, the Jetson Nano has a standalone single-board computer with a special CPU and GPU for supporting Edge AI. What is obvious is that the application of Edge AI and embedded systems has recently become widespread across various industries, including that of autonomous vehicles, industrial IoT, innovative healthcare, agriculture and intelligent factory [22]. More detailed information about Nvidia Jetson Nano and Google Coral USB Accelerator and a comparison between both devices can be found in Section 4 before the embedded device selection for this study.

### 2.3. Convolutional Neural Networks

A convolutional neural network (ConvNet or CNN) is a subclass of the ANNs and an algorithm of deep learning. It was developed to read digital information directly and derive meaningful information to solve problems of computer vision including image classification. With the popularity of computer vision and the success of CNNs, civil engineers and researchers have been attracted to this technology for the monitoring and condition assessment of structures. The CNN was inspired and generated based on the biological nervous system of humans, which contains neurons and connections. Similarly, each neuron in a CNN represents a processing unit, which is responsible for mathematically transforming input data to output values.

**Figure 3:**
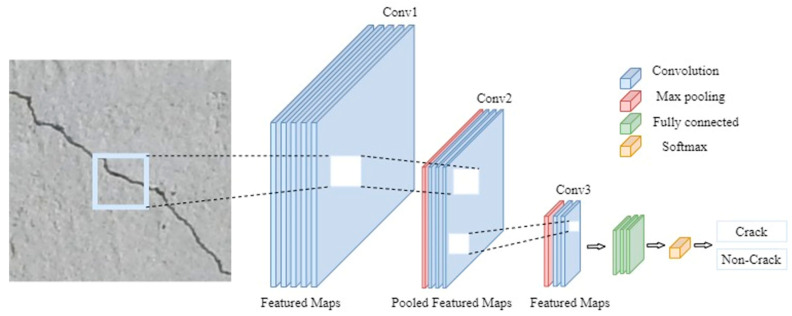
The basic architecture of a CNN.

The structure of a typical CNN contains three main types of layers, including convolutional layers, pooling layers and fully connected layers (Figure 3). The convolutional layers extract numerous features from an input image using ordered categories. For example, the first convolutional layer extracts basic data such as text, numbers, lines and edges, while the second layer extracts higher-level data such as objects or boundary regions. These extracted features are then inserted into feature maps and operated with weight matrices to perform the convolutional operation and return output values. Subsequently, the pooling layer is used to reduce the dimension of the processing unit and downsample the connections of the convolutional layer. As a result, the number of network parameters is reduced with an activation function. The pooling layer benefits the network by reducing the complexity of the feature maps and enhancing efficiency. The fully connected layer connects a single neuron from the previous layer to all the neurons in another layer. This layer plays a vital role in the classification task by comparing the extracted feature with the filter and returning the result.

LeNet-5 and AlexNet were the first two popular and successful networks for image classification [25]. LeNet-5 was built for the recognition of number images, with five layers and 60 thousand parameters [26]. It created a basic framework for researchers to enhance and generate AlexNet in 2012 with eight layers and 60 million parameters [27]. However, these networks have a limited number of layers; therefore, it takes them a long time to extract multiple features from large datasets. To deal with that problem, in 2014, GoogLeNet, also known as Inception V1, was developed with a larger number of layers and deeper architectures, consisting of nine inception modules placed sequentially with a max pooling layer in the middle [28]. This network consisted of 22 layers with four different types, namely convolutional, pooling, fully connected and softmax layers [29]. In comparison with AlexNet, its number of parameters is much lower (approximately twelve times lower) whereas the accuracy is slightly higher when training with the ImageNet database to classify 1000 object categories.

The appearance of GoogLeNet has proven that increasing the depth and width of the architecture enhances the performance of the network. However, employing deeper networks also has drawbacks such as the loss of the feature or data when the information is transferred through each layer. Consequently, the network’s performance is degraded when the network starts converging. A deep residual network (Resnet) has been introduced with batch normalisation and skip connections technologies which can be a solution for the degradation problem of deeper networks [30]. There are two types of skip connections, commonly used in Resnet18 and Resnet50 networks. For example, the Resnet18 network contains eight residual blocks and 18 deep layers. Each residual block of Resnet18 skips two blocks at once. On the other hand, the Resnet50 network employs the residual block to skip three layers, reducing the number of layers to 50 deep layers. Resnet models have been trained and tested on the ImageNet dataset for their new architecture and have returned an improvement in accuracy instead of degradation.

MobileNet is a lightweight deep neural network with a lower number of parameters and smaller model sizes compared with other convolutional neural networks. It was developed to meet the demand for application computer vision and object detection on robotic, unmanned aerial vehicles or self-drive vehicles. MobileNet V2 consists of 53 deep layers, 32 convolutional layers and 19 inverted residual bottleneck layers [31]. The state of the art in deep learning networks is MobileNet V3, with a small and a large version which represent the targeting of low and high resources, respectively. MobileNet V3 has been generated with the combination of a platform-aware neural network architecture search (NAS) and the NetAdapt algorithm [32].

As confirmed by various sources, including a recent book chapter [33], the six aforementioned convolutional networks are selected for this study due to their small and lightweight architectures as well as to provide a good balance between performance and efficiency. However, Resnet50 is an exception due to its slightly deeper and heavier architecture, and it is thus categorised between small and large models. Table 2 summarises all primary information of the networks used in this research. Another reason for choosing these small models is because of the relatively small number of parameters, which can be more easily accommodated into embedded systems. In addition, larger models typically require a larger dataset for training purposes in order to avoid overfitting.

## 3. Image Data Preparation

For this research, public datasets have been utilised for concrete crack detection [34]. The datasets contain four subsets, including Original (Orig), Salt and Pepper (SP), Motion Blur (MB) and Combination (Comb). In a realistic scenario, capturing images often encounters difficulties such as an out-of-focus condition or a dirty lens which can negatively affect the quality of image. To simulate these various aberrations, the original image dataset was augmented with three noise patterns: SP, MB and a combination of SP and MB. SP noise introduces random pixel disturbances, simulating scenarios where images might be affected by sensor noise, an incorrect digital ISO setting or transmission errors. On the other hand, MB noise mimics conditions where the camera or object is in motion during image capture, reflecting challenges in real-time inspection.

**Figure 4:**
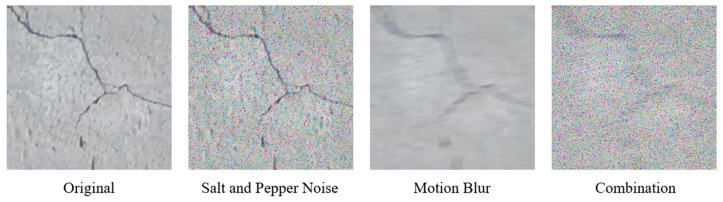
Image data sources.

The Orig dataset contains 3000 images of concrete surfaces with a size of 256 by 256 pixels. From this, the SP dataset was generated with a noise density of 6% to simulate the difficult conditions of environmental effects on the image. On the other hand, the MB dataset was developed with the motion’s length and angle of 20 pixels and 11 degrees, respectively. These parameters for the creation of the SP and MB datasets were chosen for consistency with other studies the authors are working on, but there is nothing particularly special about them and alternative values can be used. The Comb dataset is the combination of the two effects, SP and MB. Example images from the four datasets are illustrated in Figure 4 below. For the training and validation, all datasets were split into two subfolders, including training and validation, with an 80:20 ratio.

**Figure 5:**
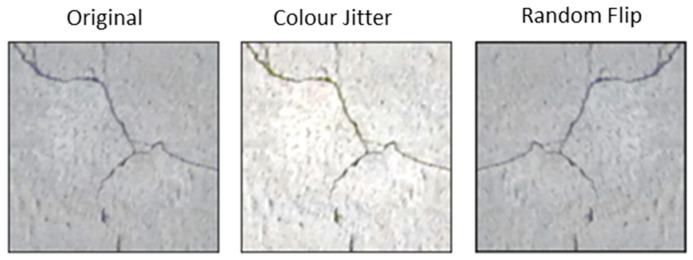
Image augmentation examples.

Before the training process, each dataset was augmented with transformations, including random crop, colour jitter and random flip. The purpose of this random image augmentation was to generalise the existing dataset without having to collect more data. As a result, the generated model was more generally applicable to field inference/prediction. The original size of the images was 256 by 256 pixels, which is a de facto standard initial resolution to facilitate the comparison of several different image classification models that potentially use different image resolutions, e.g., Resnet uses 224 × 224 image resolution, while AlexNet uses 227 × 227 image resolution. As a result, when training or validating against a specific image classification model, each original 256 × 256 image was randomly cropped to the specific size required by the model of interest. To make the dataset more generic, the image was randomly flipped vertically and/or horizontally. Additionally, the characteristics of the image, including brightness, contrast and saturation, were adjusted with a coefficient of 0.2. Figure 5 below shows the demonstration of the image augmentation.

The utilisation of a diverse concrete crack dataset with various noise patterns and augmentation algorithms significantly enhanced the generalisation of the models by exposing them to a broader range of variations and distortions that mimic real-world conditions. As a result, the training model was able to develop more invariant and robust features, enabling it to classify previously unseen image data more effectively.

## 4. Research Methodology

This research employs the following six transfer learning image classification models to detect cracks in concrete surfaces: Resnet18, Resnet50, GoogLeNet, MobileNetV2 and MobileNetV3-Small/Large. The justification for employing these specific models was discussed in Section 2, i.e., being small models, they are more appropriate for implementation in resource-constrained embedded devices and are less likely to overfit to the data during training. These models have been pre-trained with ImageNet using more than 1,200,000 images and 1000 object classes [28,30,31,32,35]. When using transfer learning to customise these models to the crack datasets, we use two mini-batch sizes of 16 and 32 images and repeat the training ten times to determine the stability and robustness of the model. There are two main stages: choosing a suitable type of transfer learning and training each network with the various datasets (Orig, SP, MB and Comb—see Section 3 for details of the datasets).

**Figure 6:**
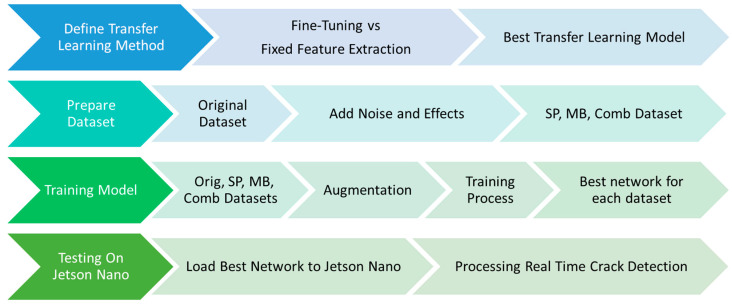
Methodology of the paper.

Figure 6 shows the methodology of the paper. In the first stage, two types of transfer learning are considered in this paper. The first method is the fixed feature extraction method. In the fixed feature approach, the original fully connected final layer of the pre-trained networks with an ImageNet dataset containing 1000 object classes is replaced by a new fully connected layer with only two categories, crack and non-crack/base. The rest of the layers remain the same. On the other hand, fine-tuning, the second transfer learning method, also utilises the pre-trained model, but the weights of all layers in the pre-trained model are optimised and updated during training. Additionally, a new fully connected final layer is added to the model to align with the target of the two categories (crack and non-crack/base). This approach enables the model to adapt its learned features to better suit the specific dataset.

The default hyperparameters for the training are shown in Table 3 below. Using a single set of hyperparameters was intentional to maintain a controlled test setup and to isolate the impact of dataset variations and augmentations on classification performance. Choosing the most suitable type of transfer learning network for the concrete crack dataset is carried out on two networks, Resnet18 and Resnet50, using the Orig dataset. The second stage of this paper involves training the rest of the networks with each of the datasets. Running various networks with a high number of epochs requires a powerful computing machine. Therefore, the NVIDIA DGX Station A100 was employed for training because it contains multiple GPUs (GPU: Graphical Processing Unit), which can accelerate training.

The process of evaluating the efficiency of a network requires four common metrics, which can measure the performance of image classification, including accuracy, precision, recall and F1-scores. These metrics are calculated based on the retrieved and relevant elements from the predictions of the networks. Precision is the proportion of crack predictions made by the model that were actually cracks. On the other hand, recall is the proportion of actual cracks that were correctly predicted by the model. F1-scores are the combination between precision and recall and are usually used for comparing the performance of two classifiers.

(1) $$Accuracy=\frac{True\;Positive+True\;Negative}{True\;Positive+True\;Negative+False\;Positive+False\;Negative}$$

(2) $$Precision=\frac{True\;Positive}{True\;Positive+False\;Positive}$$

(3) $$Recall=\frac{True\;Positive}{True\;Positive+False\;Negative}$$

(4) $$F1-\mathrm{score}=2*\frac{Precision*Recall}{Precision+Recall}$$

**Figure 7:**
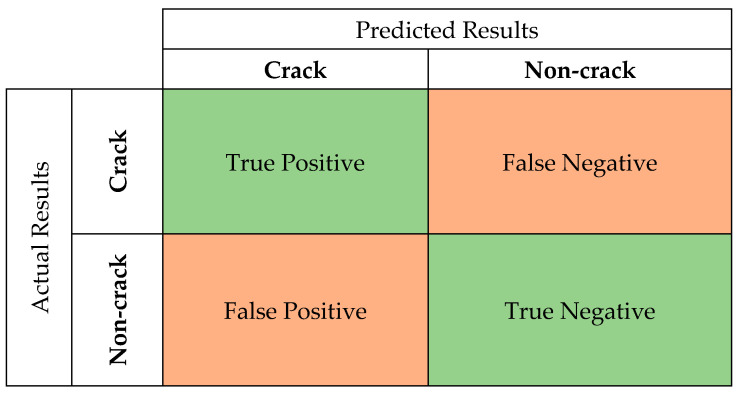
Confusion matrix for evaluating the performance of networks.

Figure 7 demonstrates the confusion matrix, which is used for determining the above performance parameters. True Positive (TP) indicates that both the predicted and actual results are crack images, while True Negative (TN) indicates that both the predicted and actual results are non-crack images. On the other hand, False Positive (FP) and False Negative (TN) represent a difference between the predicted and actual results. In particular, FP represents the prediction that the image is a crack when the image actually does not contain a crack, and False Negative (FN) represents the prediction that an image is not a crack when the image actually does contain a crack.

Several aspects, including performance, model compatibility, power consumption and storage, were evaluated in the comparison between the Nvidia Jetson Nano and the Google Coral USB Accelerator to select a suitable embedded device. The Jetson Nano is an individual device equipped with a 128-core Maxwell GPU and a quad-core ARM Cortex-A57 CPU, offering 472 GFLOPS (GFLOPS: Giga Floating Point Operations Per Second) of computational power for running diverse neural networks, including floating-point models of FP32, FP16 and int8. In contrast, the Coral USB Accelerator is a dependent device with Google’s Edge TPU with a high efficiency of 4 TOPS (TOPS: Tera Operations Per Second), optimising only int8 quantised models. As a result, the Jetson Nano is more suitable for complex tasks, model development, multi-tasking and inference compared to the Coral USB Accelerator. Additionally, the Nano supports a 64-bit LPDDR4 RAM memory card and external storage capabilities via microSD slot, features that are not available on the Coral USB device.

**Figure 8:**
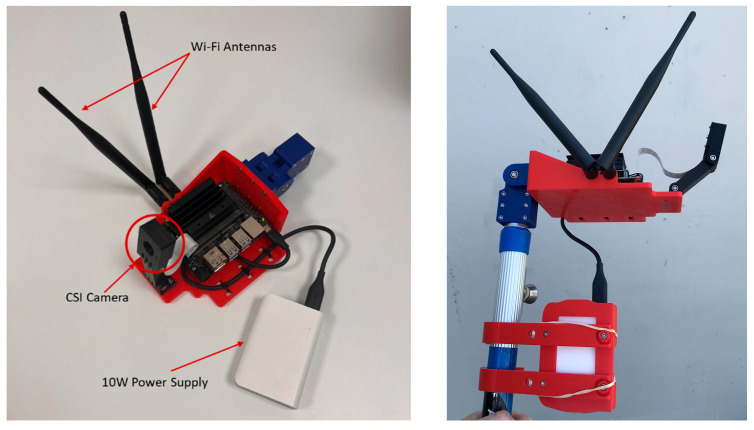
Nvidia Jetson Nano crack detector plan view (**left**) and on extension pole (**right**).

The Nvidia Jetson Nano was finally selected as the embedded Edge AI device to test the network because the device efficiently supports popular AI frameworks and models and is inexpensive. It is also more beneficial than other embedded devices, such as the Coral USB Accelerator from Google, which is not as powerful. The Jetson Nano is attached with a Camera Serial Interface (CSI) camera and a 64 GB external SD card. Since there is typically no main power available during field inspections, the device is powered by an external power bank via a micro-USB connector at 5 V/2 A maximum. Additionally, the device is also designed to adopt a power source via barrel jack at 5 V/4 A using an adapter if main power is available. To connect the Jetson Nano to a management computer, a Wi-Fi module was installed with two antennas for transferring and receiving signals. After identifying the best CNN for each dataset, the associated weights were loaded into the Jetson Nano to perform real-time detection with real crack images taken with the CSI camera. After attaching all necessary accessories, the Jetson Nano was mounted on a 3D-printed holder and connected to an extension pole, which helped assessors draw closer to a concrete surface of interest (Figure 8).

## 5. Results and Analysis

This section describes the performance of each transfer learning model. Firstly, the comparison of two transfer learning networks, fixed feature extraction and fine-tuning, and the choice of the most suitable one are undertaken using the Orig dataset. Following that, the training process for all CNNs is conducted with four types of datasets (Orig, SP, MB, Comb) and two different batch sizes.

### 5.1. Comparison Between Fine-Tuning and Fixed Feature Extraction Networks

**Figure 9:**
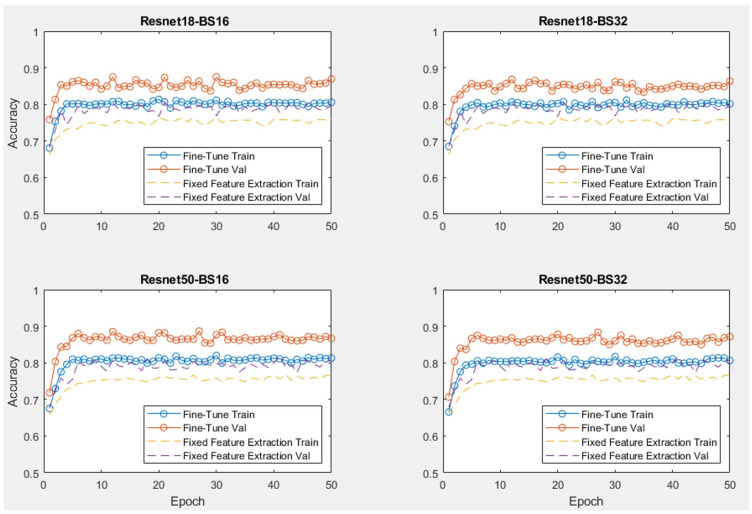
Training results of two transfer learning models.

Figure 9 demonstrates the training process of two transfer learning methods, fine-tuning and fixed feature extraction. Both methods were tested with two neural network models (Resnet18 and Resnet50). The training process was conducted with the Original dataset (Orig) and two different batch sizes, 16 and 32 units. The hyperparameters for both methods were set with a learning rate of 0.001, momentum of 0.9, step size of 7 and gamma of learning rate scheduler of 0.1; these parameters are different to the real training models. It is shown in the plot that both models of CNNs are stable and start convergence after the first 10 epochs. The accuracy of the fine-tuning models is always higher than the fixed feature extraction models by approximately 7–8%. This is expected because the weights of all the layers can be optimised with fine-tuning, compared to just the weights of the final fully connected layer with fixed feature extraction.

**Figure 10:**
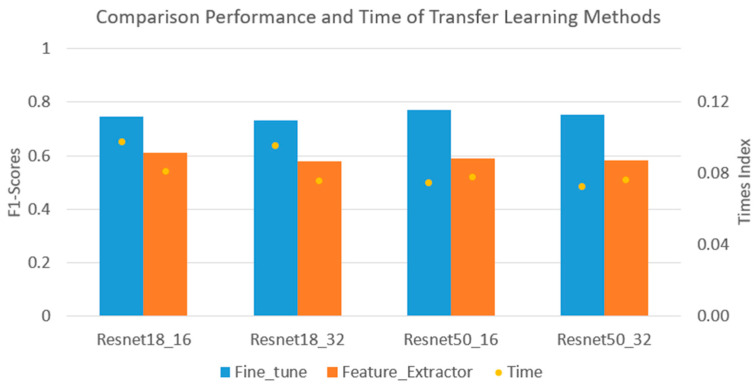
Comparison of performance and time of each transfer learning network.

Table 4 and Figure 10 illustrate the performance of each transfer learning model. The performance of the fine-tuning models for all CNNs achieved greater than 85% accuracy, with a lower accuracy of approximately 78% for the fixed featured extraction models. Furthermore, the training times of all the cases were quite similar, approximately 12–13 min, except for those of the Resnet50 networks with the fine-tuning method. These networks took around 17.2 min for training, which is significantly higher compared to the other networks. The Time Index (EI) value shows the relationship between the performance and computing time of each model. The model with a higher EI coefficient might have improved performance with less training time. It is shown in Figure 10 that the EI values of all the fine-tuning models are higher than the other models in Resnet18 networks. However, with the Resnet50 networks, these values are quite similar for both transfer learning methods. As a result, transfer learning with the fine-tuning model has shown the benefits of the training process with higher performance than the fixed feature extraction method.

### 5.2. Training with Different Datasets

#### 5.2.1. Original Dataset

**Figure 11:**
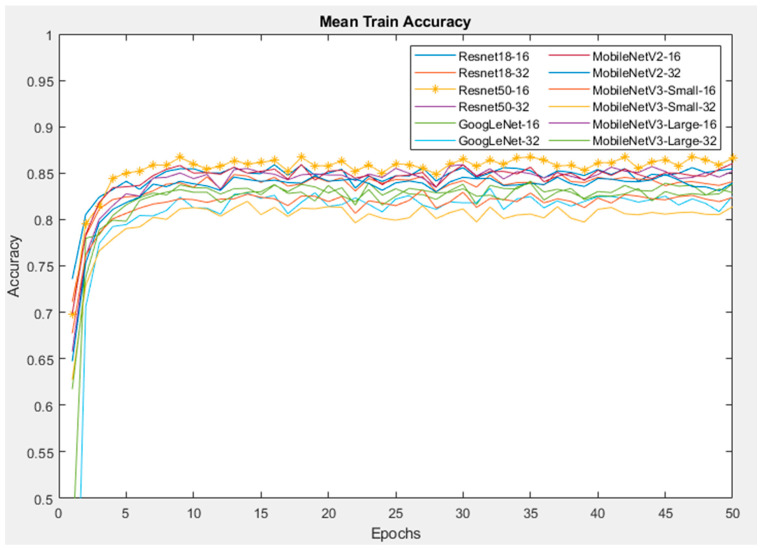
Training accuracy of CNNs with Orig dataset.

In this section, six CNNs were trained with the fine-tuning method of transfer learning. The training was repeated ten times with the Orig dataset. Figure 11 illustrates the mean training accuracy of all networks, which are similar between each run time. Furthermore, the training shows that the Resnet50 model with a batch size of 16 reaches a maximum training accuracy of approximately 85% before the first ten epochs and the accuracy then remains stable until the end. On the other hand, the MobileNetV3-Small network reaches its peak of training accuracy at 80%, which is lower than the other networks.

**Figure 12:**
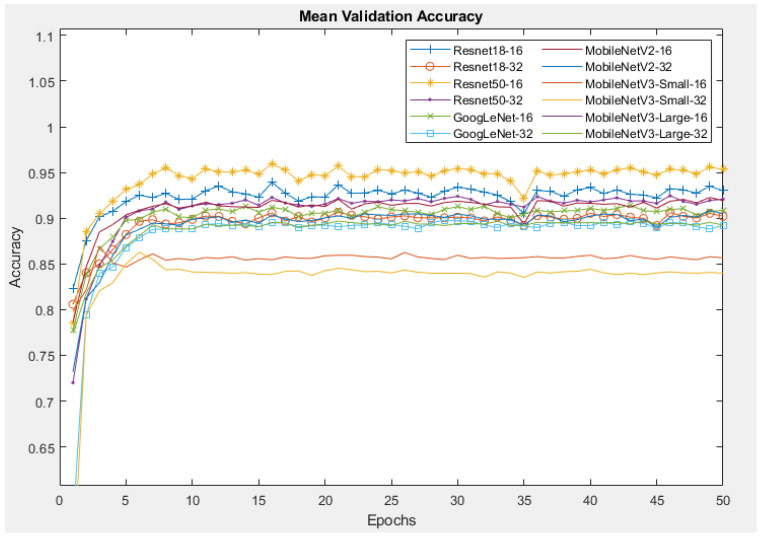
Mean validation accuracy of CNNs with Orig dataset.

Figure 12 demonstrates the mean accuracy of all models during the validation process. For all other models except the MobileNetV3-Small, the validation accuracy reaches a peak of more than 90%, which is higher than the training accuracy by 5%, and almost remains at that value until the end. A possible reason for this is that the training was conducted using data augmentation to generalise/regularise the model and prevent overfitting, whereas no data augmentation was performed on the validation images. Resnet50 with a batch size of 16 provides the highest validation accuracy of 96%, while the lowest accuracy of 83% belongs to MobileNetV3_Small with a batch size of 32.

**Figure 13:**
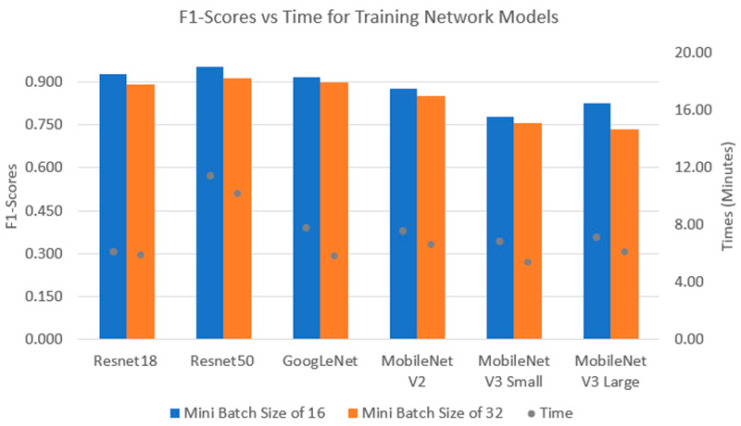
F1-scores vs. computational time of CNNs with Orig dataset.

The performance indices and computational times of the CNNs can be found in Figure 13. For a batch size of 16, Resnet50 provided the highest F1-score of 95%, followed by Resnet18, GoogLeNet and MobileNetV2. MobileNetV3-Small and large networks yield the lowest F1-scores with a similar computational time compared to other networks. For a batch size of 32, Resnet50 achieves similar F1-scores to Resnet18 and GoogLeNet. On the other hand, Resnet50 took 5 more minutes for training time than the other CNNs.

#### 5.2.2. Salt and Pepper and Motion Blur Datasets

In this section, the CNNs were trained with augmented SP and MB datasets ten times each. The training accuracy lines of each network are divided into two parts. The training accuracy trajectories of all the networks are similar, increasing up to their maximum accuracies during the first ten epochs. Then, their accuracies remained around their respective stable values without any significant fluctuations until the training process ended.

**Figure 14:**
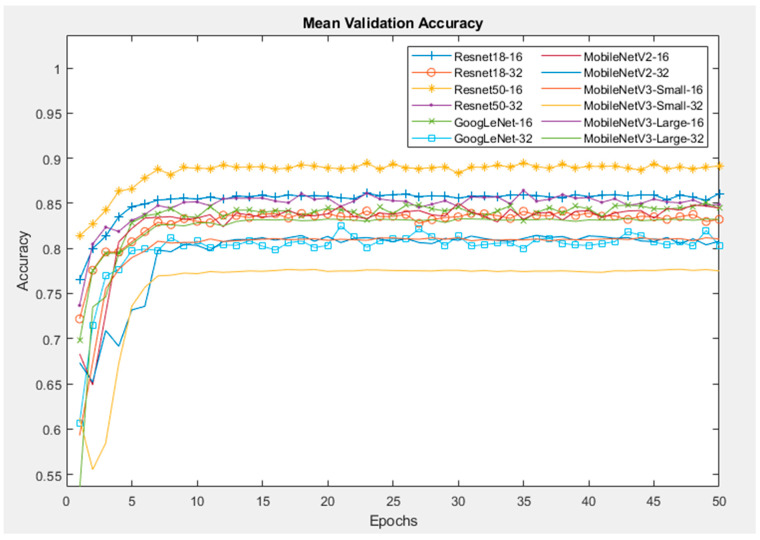
Mean validation accuracy of CNNs with SP dataset.

The mean validation accuracies of the CNNs with the SP dataset are shown in Figure 14. Similar to the Orig dataset, the Resnet50 and Resnet18 networks have the highest validation accuracies of 88% and 84%, respectively. The MobilenetV3-Small network has the lowest accuracy.

**Figure 15:**
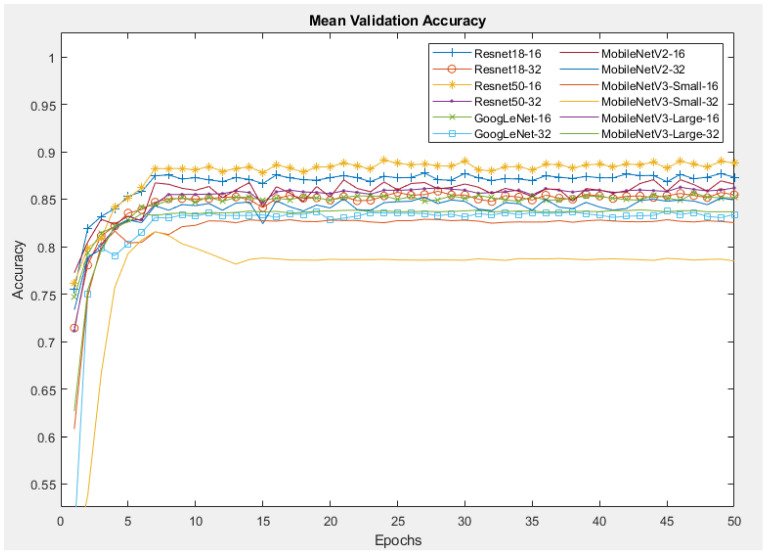
Mean validation accuracy of CNNs with MB dataset.

Similarly, with the MB dataset, as illustrated in Figure 15, the Resnet networks with a batch size of 16 yield the highest accuracy of around 87%, followed by MobineNetV2 with a batch size of 16, yielding 85% accuracy. The network with the lowest validation accuracy is MobileNetV3-Small, with their percentage just above 77%.

**Figure 16:**
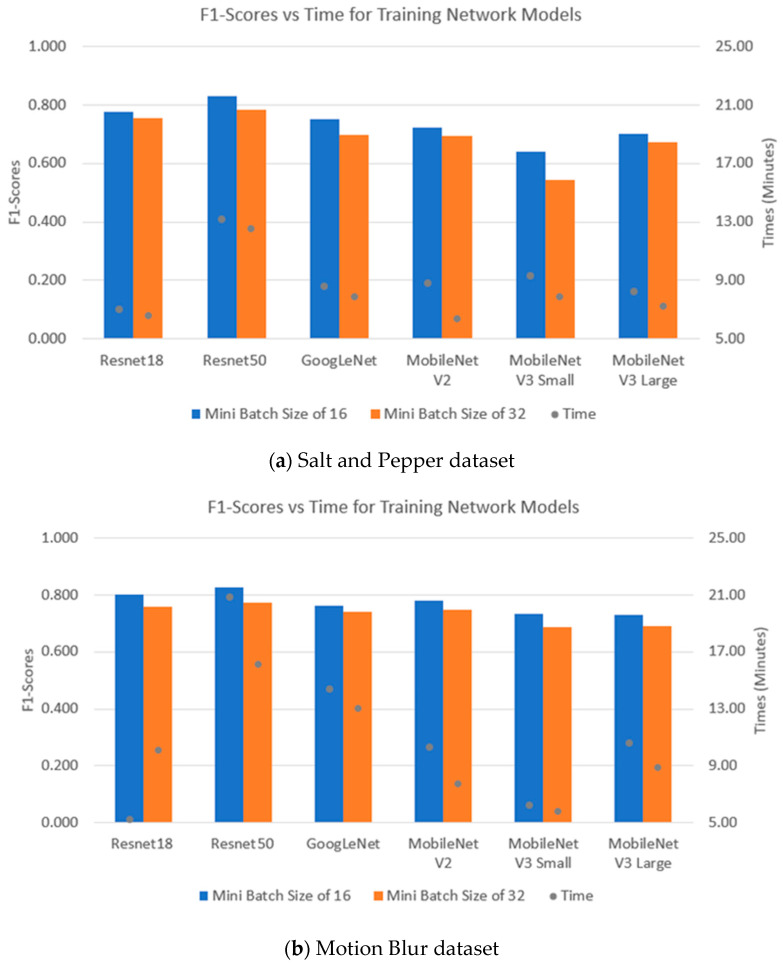
Performance indices of CNNs with different datasets: SP (**top**) and MB (**bottom**).

Figure 16 illustrates the F1-scores and training times of the CNNs for the SP and MB datasets. The CNNs work better with the MB dataset, as illustrated by their higher F1-scores compared to the SP dataset. As a result, the fine-tuning method of transfer learning with these CNNs is more compatible with the MB dataset than with the SP dataset. In detail, for the MB dataset, Resnet50 is the best model and has the highest F1-score of about 82% with a computational time of 21 min, followed by Resnet18 with an F1-score of 80% and training time of 5 min. GoogLenet, MobileNetV3 and MobileNetV2 achieved almost the same value of approximately 70% for their F1-score. With the SP dataset, Resnet50 is also the best network with the highest F1-score, but it also has the highest training time. Furthermore, the MobileNet network achieved the weakest performance, but its training times are quite similar to GoogLeNet and Resnet18. The results from both datasets showed that Resnet50 with a batch size of 16 always achieved the highest performances. Furthermore, CNN models with a batch size of 16 always achieved higher F1-scores than the same models with a batch size of 32.

#### 5.2.3. Combination Dataset

**Figure 17:**
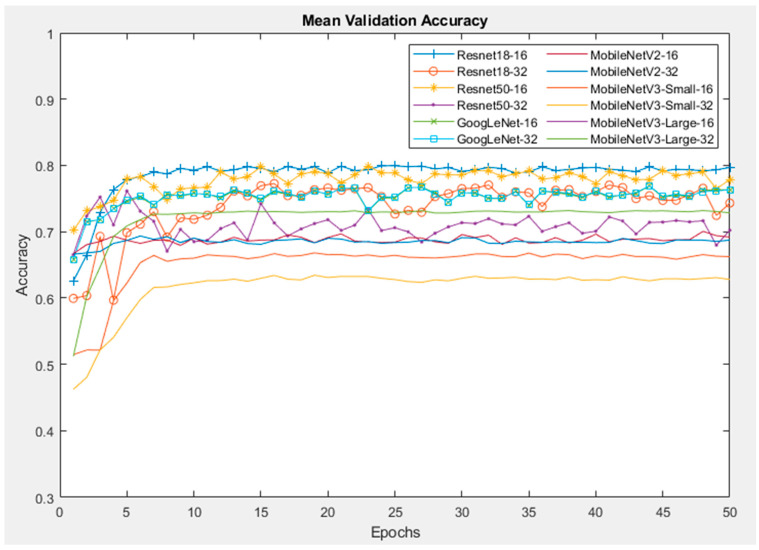
Mean validation accuracy of CNNs with Comb dataset.

**Figure 18:**
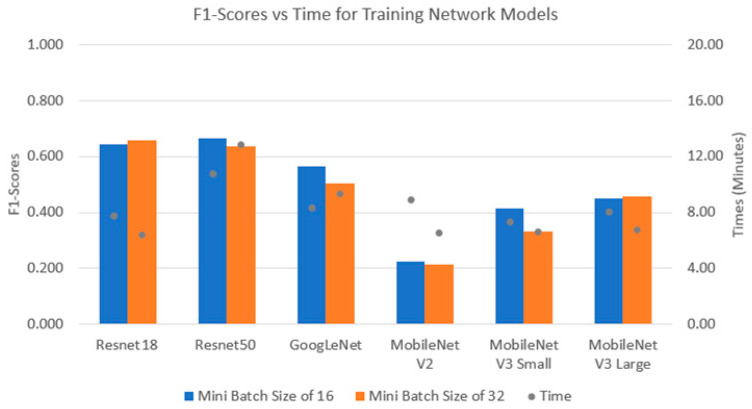
Performance indices of CNNs with Comb dataset.

Figure 17 and Figure 18 illustrate the mean validation accuracy and F1-score/time performance of the CNNs, respectively, with Comb dataset. It can be seen that the computational time of each network in this dataset is no longer than that of the three other datasets, whereas the diversity of the dataset is increased by adding the SP noise and blur images into it, as well as the augmentations for the images. It is shown that the network with highest accuracy for this dataset is Resnet18 with batch sizes of both 16 and 32. Then, it is followed by the Resnet50 and GoogLeNet models. On the other hand, the MobileNet networks show a significantly poorer performance than the other networks, but their performances are stable and mostly flat without any significant fluctuations with an increasing number of epochs. However, when considering F1-scores, Resnet50 is the model with highest F1-score, followed by Resnet18 and GoogLeNet. Furthermore, the mean training time of Resnet50 was higher than all other networks.

### 5.3. Performance on Jetson Nano Crack Detector

After determining that the best model for the Orig dataset was Resnet50, the weights for this model were loaded into the Jetson Nano for inference testing with images of concrete surfaces captured in real time using a Raspberry Pi v2 8MP CSI camera attached to the Nano. The test was performed in the field to detect cracks on the concrete walls of an underpass structure (culvert). Additionally, in the laboratory, the Jetson Nano-based inference engine was also assessed with cracked concrete cylinders using different viewing angles. The test returned the prediction for the image from two candidate labels: “crack” and “base”. “Crack” means that the image was detected with one or more cracks, while “base” indicates that the image does not contain a crack.

**Figure 19:**
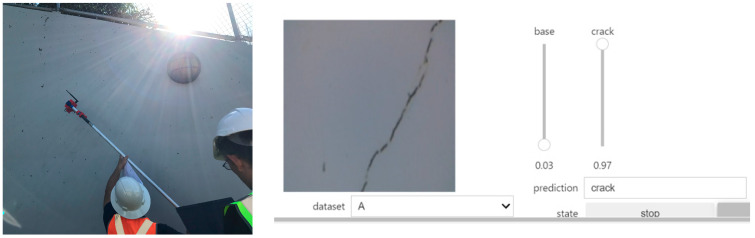
Example of real-time crack detection by Nvidia Jetson Nano in the field.

**Figure 20:**
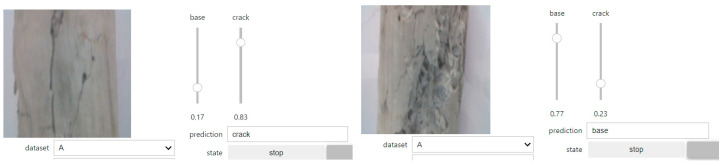
Example of real-time crack detection by Nvidia Jetson Nano in the laboratory.

In the test, the Jetson Nano was linked to the inspectors’ laptop via Wi-Fi, which required both devices to be connected to the same network. As a result, the assessor was able to control and visualise the real-time predictions of the Jetson Nano. Figure 19 and Figure 20 show the Jetson Nano-based inference engine in action in the field and in the laboratory, respectively. The Jetson Nano-based inference engine predicted cracks in images with high accuracy. However, for some difficult concrete cracks or spalling, the device was unable to detect the aberration and gave the wrong decision. This problem might be solved by training against a much larger dataset and using more advanced models in future works.

## 6. Discussion and Comparison

### 6.1. Discussion

With respect to the training and validation of the six sample compact CNN models, it was found that Resnet50 had a superior performance in the Orig dataset, which is attributed to its deeper architecture and residual learning that enables the model to learn complex patterns effectively. Similarly, for the SP and MB datasets, the best classification performance also belonged to the Resnet50 model, which highlights its robustness to noise and distortion because of its residual connection and depth. Resnet18 demonstrates competitive performance on the Comb dataset with its highest validation accuracy. This suggests that its efficient architecture balances capacity and computational demand well. MobileNetV3-Small, despite its efficiency, underperforms on the more challenging datasets (i.e., those with added noise), indicating its limited ability to handle significant distortions.

All six selected CNN models illustrated varied strengths and weaknesses across the difficult and complex datasets under noisy conditions and augmentations. Resnet50 proved its robustness and accuracy, excelling in all scenarios due to the depth of its architecture and residual connections, which allow the model to extract crack features effectively under challenging simulated environments. Following that, Resnet18, with its shallower architecture and residual connections, provided a good performance on complex datasets while requiring a slightly lower computational cost, making it an excellent alternative selection.

GoogLeNet showed reliability due to its multi-scale feature extraction through the inception module, enabling it to handle noise such as SP and MB reasonably well, though its moderate depth made it less effective than the Resnet models on highly complex datasets. The MobileNet models offered a balance between efficiency and performance, handling clean datasets effectively but showing some limitations in robustness under augmented and noisy conditions. MobileNetV3-Small struggled significantly with the distorted datasets due to its smaller representational capacity, while it was the most efficient in terms of computational requirements.

### 6.2. Comparison with Alternative Studies

A study investigated the assistance of the BRISQUE threshold-based method for enhancing classification performance on concrete cracks [36], which shares a similar diversity dataset with this study. The term BRISIQUE stands for Blind/Referenceless Image Spatial Quality Evaluator, meaning the evaluation can be conducted without the original, undistorted image. That study generated artificial images based on the original dataset with the addition of Gaussian noise and Gaussian blur. Then, the BRISQUE algorithm was applied to the dataset to produce a score which represented the image’s quality. The low-quality image contained a high proportion of noise and blur, which achieved a high BRISQUE score. On the other hand, a low BRISQUE score (below 45) represents high-quality images. The study found that the correlation between the BRISQUE score and classification performance was inversely proportional, which was also found in this paper with a low-quality dataset that returned weak classification performance. In both studies, the best performance of the classification task was found on the model which was trained on the clean and original dataset.

In the case of the study using STELM reviewed earlier, it was found that their TL could effectively perform crack classification tasks [19] and have some benefit of using double-step transfer learning with two different Resnet modes. However, the effects of the image quality under severe environments and real conditions on the classification performance were not investigated. In contrast, our TL method was tested against images affected by noise. This method is also computationally efficient, making it more feasible to deploy Edge AI devices with limited computational resources. This efficiency is critical in real-world applications where quick inference and resource constraints are significant considerations. It can be concluded that our methodology can provide robust performance in practical conditions, benefiting from the generalisation capabilities of the pre-trained model while being adaptable to the specific needs of concrete crack detection.

## 7. Conclusions

This paper presents a methodology and comparison of several transfer learning models for detecting cracks on concrete surfaces and targeting implementation onto Edge AI devices such as those in the Nvidia Jetson family. Six convolutional neural networks were selected for the test, namely Resnet18, Resnet50, GoogLeNet, MobileNetV2 and two versions of MobileNetV3 (one large and one small). To generalise the applicability of the models, the Orig dataset and its augmented variations (SP, MB and Comb) were utilised for the training and validation processes. Firstly, the transfer learning methods were investigated and a comparison between the fine-tuning and fixed feature extraction methods was conducted. Then, the best-performing transfer learning method, fine-tuning, was chosen and applied to all of the CNNs, followed by training on four different datasets (Orig, SP, MB and Comb).

The primary results of this paper are described by the following points:

- Transfer learning with the fine-tuning method and specific hyperparameters is more reliable and efficient rather than the fixed feature extraction method.
- In the Orig dataset, the Resnet50 network with a batch size of 16 showed the highest accuracy and F1-score. Other CNNs, especially MobileNet V3 Small, had a weaker performance than Resnet50.
- When considering augmentation with the SP and MB datasets, Resnet50 showed its strength and reliability. The validation accuracy of the Resnet50 model for both datasets was around 82%.
- Resnet18 returned the highest validation accuracy, whereas the highest F1-score belonged to Resnet50 when dealing with the most complicated dataset (Comb) and augmentation. It can be seen that the time saved was not significant between the large and small networks, very likely due to the complexity of the dataset.

This paper shows an important comparison between six transfer learning networks and complicated datasets with augmentations with a focus on small batch sizes that suit the computation capacity of Edge AI devices. It can be seen that, apart from GoogLeNet in the MB and Comb datasets, all other networks and datasets training with a batch size of 16 achieved a higher accuracy and performance index than when implementing a batch size of 32. However, the training time between the two batch sizes in each network were not much different. The networks were able to run successfully on a Jetson Nano-based inference engine in terms of detecting cracks in real time on the concrete surfaces in the field and laboratory environments.

In summary, the results have shown Resnet50 to be the most robust and reliable transfer learning model among the six networks investigated in this study. Future work should focus on tuning the hyperparameters for transfer learning and improving the performance of smaller models such as Resnet18 or MobileNet because they are more lightweight and might be better suited for inexpensive embedded devices. In the longer term, we plan to investigate object detection and the semantic segmentation of crack images using embedded devices to locate cracks using bounding boxes or to characterise the size and shape of each crack for defect severity assessment.

## Figures and Tables

**Table 1:** Computer vision techniques.

Objective	Computer Vision Technique
Determining the class(es) of object(s) in an image	Image Classification
Placing a bounding box around each detected object in an image	Object Detection
Determining the exact pixel boundaries of each detected object in an image	Semantic Segmentation

**Table 2:** Summary information of the six CNNs.

Network	No. of Layers	Parameters (Millions)	Size of Model (MB)	Image Size (Pixels)
Resnet18	18	1.6	44.8	224 × 224
Resnet50	50	25.6	94.4	224 × 224
GoogLeNet	22	7	26.7	224 × 224
MobileNetV2	53	3.5	9.1	224 × 224
MobileNetV3 Small	16	2.9	6.2	224 × 224
MobileNetV3 Large	20	5.4	17	224 × 224

**Table 3:** Hyperparameters for transfer learning.

Parameter	Value
Initial learning rate	1 × 10^−4^
L2 regularisation	0.005
Momentum	0.9
Optimisation algorithm	SGDM
Epochs	100
Learning rate scheduler step	5
Gamma for learning rate scheduler	0.001

**Table 4:** Summary performance index of transfer learning networks.

Transfer Learning Type	CNN	Batch Size	Accuracy	Precision	Recall	F1-Score	Time (Min)
**Fine-Tuning**	Resnet18	16	0.8517	0.98	0.60	0.75	12.7
		32	0.8467	0.96	0.59	0.73	12.7
	Resnet50	16	0.8628	0.99	0.63	0.77	17.2
		32	0.8576	0.99	0.61	0.75	17.2
**Fixed Feature Extraction**	Resnet18	16	0.7849	0.84	0.48	0.61	12.5
		32	0.7849	0.86	0.44	0.58	12.7
	Resnet50	16	0.7878	0.88	0.45	0.59	12.7
		32	0.7878	0.90	0.43	0.58	12.7

## Data Availability

The raw data supporting the conclusions of this article will be made available by the authors on request.
